# Novel Plasmid-Mediated Colistin Resistance Gene *mcr-3* in *Escherichia coli*

**DOI:** 10.1128/mBio.00543-17

**Published:** 2017-06-27

**Authors:** Wenjuan Yin, Hui Li, Yingbo Shen, Zhihai Liu, Shaolin Wang, Zhangqi Shen, Rong Zhang, Timothy R. Walsh, Jianzhong Shen, Yang Wang

**Affiliations:** a Beijing Advanced Innovation Center for Food Nutrition and Human Health, College of Veterinary Medicine, China Agricultural University, Beijing, China; b The Second Affiliated Hospital of Zhejiang University, Zhejiang University, Hangzhou, China; c Department of Medical Microbiology and Infectious Disease, Institute of Infection and Immunity, Heath Park Hospital, Cardiff, United Kingdom; Indiana University Bloomington

**Keywords:** *Aeromonas*, colistin resistance, *Enterobacteriaceae*, *mcr-3*, public health

## Abstract

The mobile colistin resistance gene *mcr-1* has attracted global attention, as it heralds the breach of polymyxins, one of the last-resort antibiotics for the treatment of severe clinical infections caused by multidrug-resistant Gram-negative bacteria. To date, six slightly different variants of *mcr-1*, and a second mobile colistin resistance gene, *mcr-2*, have been reported or annotated in the GenBank database. Here, we characterized a third mobile colistin resistance gene, *mcr-3*. The gene coexisted with 18 additional resistance determinants in the 261-kb IncHI2-type plasmid pWJ1 from porcine *Escherichia coli*. *mcr-3* showed 45.0% and 47.0% nucleotide sequence identity to *mcr-1* and *mcr-2*, respectively, while the deduced amino acid sequence of MCR-3 showed 99.8 to 100% and 75.6 to 94.8% identity to phosphoethanolamine transferases found in other *Enterobacteriaceae* species and in 10 *Aeromonas* species, respectively. pWJ1 was mobilized to an *E. coli* recipient by conjugation and contained a plasmid backbone similar to those of other *mcr-1*-carrying plasmids, such as pHNSHP45-2 from the original *mcr-1*-harboring *E. coli* strain. Moreover, a truncated transposon element, Tn*As2*, which was characterized only in *Aeromonas salmonicida*, was located upstream of *mcr-3* in pWJ1. This ΔTn*As2*-*mcr-3* element was also identified in a shotgun genome sequence of a porcine *E. coli* isolate from Malaysia, a human *Klebsiella pneumoniae* isolate from Thailand, and a human *Salmonella enterica* serovar Typhimurium isolate from the United States. These results suggest the likelihood of a wide dissemination of the novel mobile colistin resistance gene *mcr-3* among *Enterobacteriaceae* and aeromonads; the latter may act as a potential reservoir for *mcr-3*.

## IMPORTANCE

The emergence of the plasmid-mediated colistin resistance gene *mcr-1* has attracted substantial attention worldwide. Here, we examined a colistin-resistant *Escherichia coli* isolate that was negative for both *mcr-1* and *mcr-2* and discovered a novel mobile colistin resistance gene, *mcr-3*. The amino acid sequence of MCR-3 aligned closely with phosphoethanolamine transferases from *Enterobacteriaceae* and *Aeromonas* species originating from both clinical infections and environmental samples collected in 12 countries on four continents. Due to the ubiquitous profile of aeromonads in the environment and the potential transfer of *mcr-3* between *Enterobacteriaceae* and *Aeromonas* species, the wide spread of *mcr-3* may be largely underestimated. As colistin has been and still is widely used in veterinary medicine and used at increasing frequencies in human medicine, the continuous monitoring of mobile colistin resistance determinants in colistin-resistant Gram-negative bacteria is imperative for understanding and tackling the dissemination of *mcr* genes in both the agricultural and health care sectors.

## OBSERVATION

Since we first reported the mobile colistin resistance gene *mcr-1* in China in 2016 (1), there have been reports of *mcr-1* in *Enterobacteriaceae* isolated from animals, animal products, humans, and the environment in over 30 countries across five continents (2). More worrisome is the presence of *mcr-1* in *Enterobacteriaceae* carrying carbapenem resistance genes, such as *bla*_NDM_ and *bla*_KPC_, which would seriously compromise the treatment of infections caused by these extensively drug-resistant pathogens (2). Meanwhile, six variants of the *mcr-1* gene have been described, including *mcr-1.2* (GenBank accession no. KX236309) in KPC-3-producing *Klebsiella pneumoniae* isolated from a rectal swab of a leukemic child (3), *mcr-1.3* (NG_052861) in *Escherichia coli* from chickens in China, *mcr-1.4* (KY041856) in *E. coli* from sewage in China, *mcr-1.5* (KY283125) in *E. coli* isolated from a human urinary tract in Argentina, *mcr-1.6* (NG_052893) in *Salmonella enterica* serovar Typhimurium from a healthy human in China, and *mcr-1.7* (KY488488) in *E. coli* from sewage in China. These gene variants encode phosphoethanolamine transferase enzymes but differ from MCR-1 at a single amino acid: Gln_3_ to Leu in MCR-1.2, Ile_37_ to Val in MCR-1.3, Asp_439_ to Asn in MCR-1.4, His_451_ to Tyr in MCR-1.5, Arg_535_ to His in MCR-1.6, and Ala_214_ to Thr in MCR-1.7. Moreover, a novel plasmid-borne colistin resistance gene showing 77.3% nucleotide identity and 81.0% amino acid identity to *mcr-1* was also identified in porcine and bovine colistin-resistant *E. coli* isolates and was named *mcr-2* (4). Here, we report the discovery of another novel *mcr* variant, *mcr-3*, which coexisted with 18 additional resistance genes on a conjugative plasmid from *E. coli* of pig origin.

In a routine surveillance study of antimicrobial resistance of bacteria from farm animals in 2015, a colistin-resistant (MIC, ≥4 µg/ml) isolate, WJ1, was obtained from a fecal sample of an apparently healthy pig at a conventional farm in Shandong Province, China. Matrix-assisted laser desorption ionization–time of flight mass spectrometry (MALDI-TOF MS) analysis (Bruker Daltonik GmbH, Bremen, Germany) and 16S rRNA sequencing identified WJ1 as *E. coli*, and multilocus sequence typing (2) confirmed the sequence type as 1642. Although PCR screening assays for the presence of *mcr-1* and *mcr-2* were negative, the colistin resistance determinant could be transferred to *E. coli* strain EC600 by conjugation, with transconjugant EC600-WJ1 also exhibiting a multidrug resistance profile (Table 1). A ca. 250-kb plasmid was observed in both WJ1 and EC600-WJ1 by S1 nuclease pulsed-field gel electrophoresis analysis (data not shown), suggesting that this multiresistance plasmid, designated pWJ1, carried an unknown colistin resistance gene.

**TABLE 1:** MIC profiles of *mcr-3*-carrying *Escherichia coli* isolate WJ1, its transconjugant Ec600 + pWJ1, and recipient isolate EC600

Drug^b^	MIC (mg/liter) for *E. coli* strain:			Associated resistance gene(s) in plasmid pWJ1
	WJ1	EC600	EC600 + pWJ1	
COL	8	0.5	4	*mcr*-*3*
PB	8	0.25	4	*mcr*-*3*
CIP	128	0.0625	0.25	*aac(6′)-Ib-cr*
CHL	512	4	512	*floR*, *cmlA1*, *catB3*
STR	64	8	64	*strA*, *strB*, *aadA1*, *aadA2*
GEN	512	1	512	*aac(3)-IVa*
RIF	512	512^a^	512	*arr3*
AMC	32/16	4/2	32/16	*bla*_OXA-1_
SXT	>32/608	0.5/9.5	>32/608	*sul1*, *sul2*, *sul3*
TET	128	1	64	*tet*(A)

a EC600 is a rifampin-resistant strain used for transconjugation.

b Abbreviations: COL, colistin; PB, polymyxin; CIP, ciprofloxacin; CHL, chloramphenicol; STR, streptomycin; GEN, gentamicin; RIF, rifampin; AMC, amoxicillin-clavulanic acid; SXT, trimethoprim-sulfamethoxazole; TET, tetracycline.

**FIG 1:**
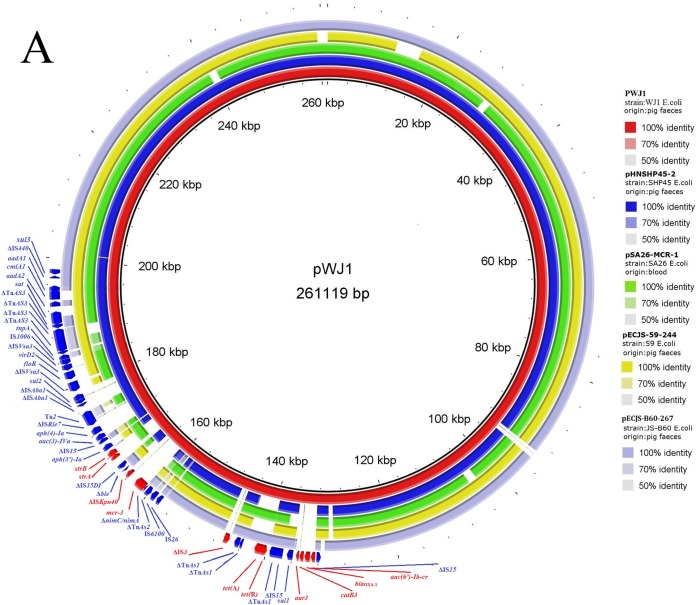

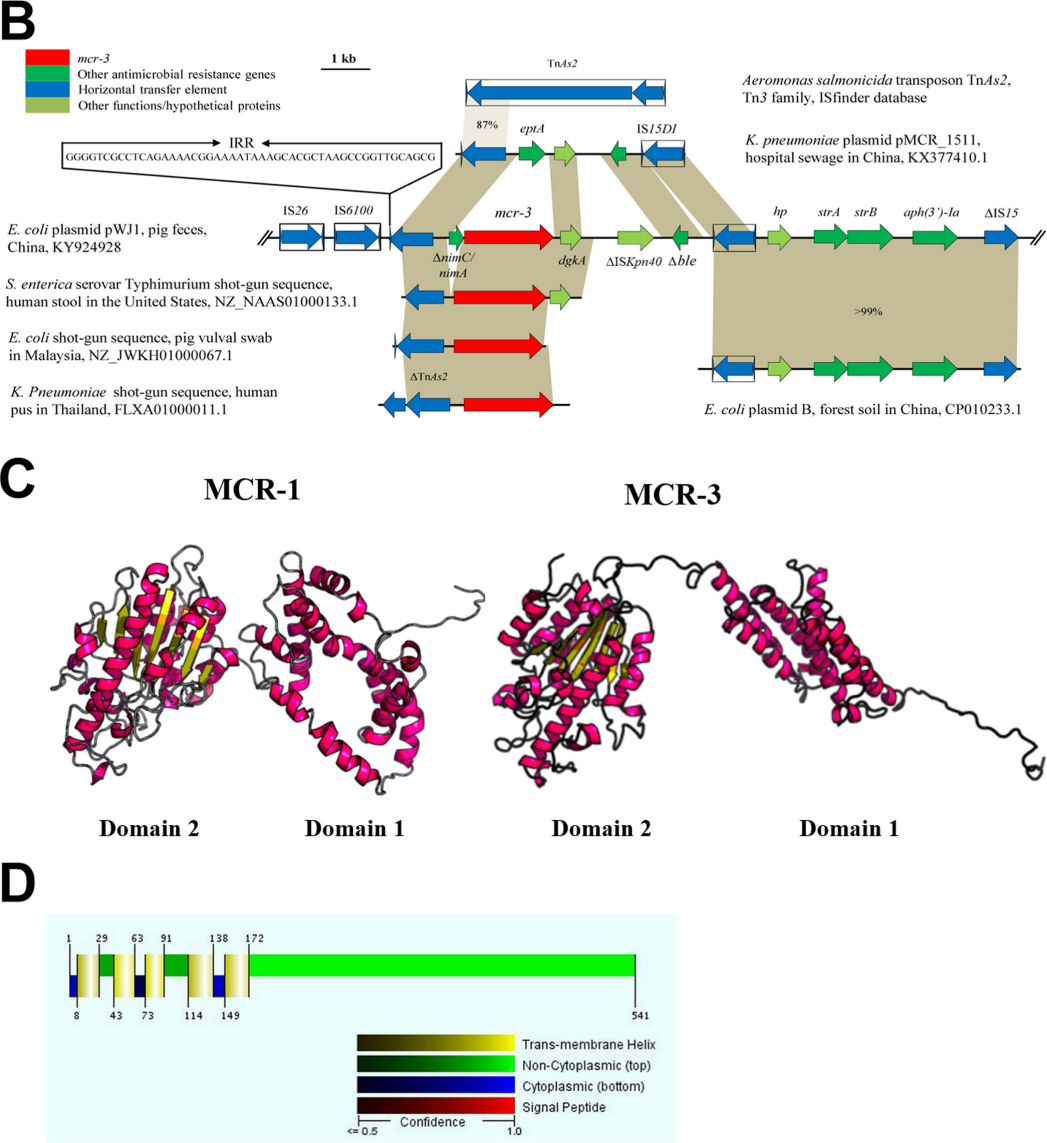
(A) BRIG analysis of the *mcr-3*-carrying plasmid pWJ1. Comparative analysis of pWJ1 with four closely related *mcr-1*-harboring plasmids from *E. coli* isolates using the BLAST Ring Image Generator (10). The concentric rings display similarity between the reference sequence in the inner ring and the other sequences in the outer rings. The various color levels indicate a BLAST result with a matched degree of shared regions, as shown to the right of the ring. (B) Comparison of the genetic environments of *mcr-3* genes in different plasmids and shotgun sequences extracted from the GenBank database. Arrows indicate the positions and directions of the genes; Δ indicates the truncated gene. Regions with >99% homology are indicated in gray shadow, with homology of >85% shown by a lighter gray shadow. (C) Structure prediction for the *mcr-3* gene product, MCR-3. Domain 1 was predicted to be a transmembrane domain, while domain 2 was predicted to be phosphoethanolamine transferase. (D) The five transmembrane α-helices predicted by the Philius transmembrane prediction server (type confidence, 0.99; topology confidence, 0.88).

pWJ1 was extracted from transconjugant EC600-WJ1 using a Plasmid Midi kit (Omega, Norcross, GA) and then sent for single-molecule real-time (SMRT) sequencing using a PacBio RSII system (Sinobiocore, Beijing, China). Plasmid assembly was performed using the hierarchical genome assembly process (HGAP) and Quiver as part of the SMRT analysis (version 2.3) using the HGAP3 protocol. Whole-cell DNA from original strain WJ1 was extracted using a Wizard genomic DNA purification kit (Promega, Beijing, China) and used as the template for whole-genome sequencing using the Illumina HiSeq 2500 system (Annoroad, Beijing, China). A draft assembly of the sequences was obtained using CLC Genomics Workbench 9 (CLC Bio-Qiagen, Aarhus, Denmark). Comparative analysis of the SMRT and HiSeq sequencing results revealed that pWJ1 was an IncHI2-type plasmid, with a size of 261,119 bp. It had a similar backbone as several *mcr-1*-carrying plasmids (Fig. 1A). For instance, a 222.3-kb section of pWJ1 (85.4% of the total sequence) exhibited high similarity to the corresponding region of the 251-kb plasmid pHNSHP45-2 from *E. coli* strain SHP45, from which the first *mcr-1* gene was identified (5). pWJ1 and pHNSHP45-2 had 12 resistance genes in common (Fig. 1A), while pWJ1 appeared to have acquired six additional resistance genes (Fig. 1A; Table 1), two of which conferred resistance to antimicrobials important for human medicine, including quinolones [*aac(6′)-Ib-cr*] and rifampin (*arr3*).

Further analysis of pWJ1 revealed a 1,626-bp putative phosphoethanolamine transferase gene, designated *mcr-3*, which exhibited 45.0% and 47.0% nucleotide sequence identity to *mcr-1* and *mcr-2*, respectively. To confirm the role of this putative polymyxin resistance gene, an 1,896-bp DNA fragment including *mcr-3* and its upstream sequence was ligated into cloning vector pUC19, yielding pUC19-*mcr-3*. This recombinant vector was further transferred to *E. coli* W3110 by electroporation. Compared with *E. coli* W3110 containing pUC19 alone, an 8-fold increase in the MIC of colistin (from 0.5 µg/ml to 4 µg/ml) was observed for *E. coli* W3110 containing pUC19-*mcr-3*, suggesting that the *mcr-3* product is responsible for the polymyxin resistance. We further screened colistin-resistant *E. coli* isolates collected from pig feces (*n* = 380) and chicken cloacae (*n* = 200) in our routine surveillance study in 2015 using the primers MCR3-F (5′-TTGGCACTGTATTTTGCATTT-3′) and MCR3-R (5′-TTAACGAAATTGGCTGGAACA-3′) and the following cycling conditions: 30 cycles of 95°C for 30 s, 50°C for 30 s, and 72°C for 45 s, followed by 1 cycle of 72°C for 7 min. The presumptive 542-bp PCR product of *mcr-3* was sent for sequencing. We detected *mcr-3* in 7 (1.8%) *E. coli* isolates from pigs only.

The deduced amino acid sequence of the *mcr-3* gene product, MCR-3, showed 32.5% and 31.7% amino acid identity to MCR-1 and MCR-2, respectively. Similarly to MCR-1 and MCR-2, the MCR-3 protein was predicted to have two domains using RaptorX (Xu group, Chicago, IL) (Fig. 1C). Domain 1 (residues 1 to 172) was predicted to contain five transmembrane α-helices (Fig. 1D), while domain 2 (residues 173 to 541) was a predicted periplasmic domain containing the putative catalytic center. When using the Swiss Model server for homology modeling, the best-fit structure in Protein Data Bank for domain 2 of MCR-3 was 4KAV, which is demonstrated by phosphoethanolamine transferase LptA from *Neisseria meningitidis* and has been previously exhibited to be the best fit for both MCR-1 and MCR-2 (1, 4).

**FIG 2:**
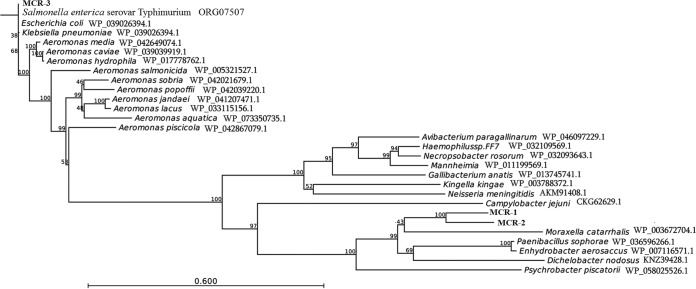
Phylogenetic tree of the deduced amino acid sequences of 28 putative phosphoethanolamine transferases from different bacterial species with MCR-3 using CLC Genomics Workbench 9 (CLC Bio-Qiagen, Aarhus, Denmark).

Compared with 28 other phosphoethanolamine transferases, including MCR-1 and MCR-2, using CLC Genomics Workbench 9, MCR-3 exhibited 99.8% to 100% amino acid identity to those found in an *E. coli* isolate from a pig in Malaysia, in *K. pneumoniae* isolates from human pus and urine samples in Thailand, and in *S. enterica* serovar Typhimurium from human stool in the United States (Fig. 2; see Table S1 in the supplemental material). This finding indicated that the *mcr-3* gene was already present in at least three *Enterobacteriaceae* species in both agricultural and clinical settings in Southeast Asia and North America. MCR-3 also showed 94.1% to 94.8% amino acid identity to proteins found in three *Aeromonas* species, including one *A. hydrophila* isolate from human peritoneal fluid and one *A. caviae* isolate from lake water in Malaysia and one *A. media* isolate of unknown origin. In addition, MCR-3 aligned closely (75.6% to 84.5% amino acid identity) with MCR-3-like sequences from eight *Aeromonas* species derived from humans, fish, water, and wetland samples from 10 countries in Asia, Europe, and North and South America (Fig. 2; Table S1). The species *A. hydrophila*, *A. caviae*, and *Aeromonas sobria* were recognized as the most common *Aeromonas* pathogens for humans (6). Furthermore, the 801-bp fragment located immediately upstream of *nimC*/*nimA*-*mcr-3* in pWJ1 exhibited 87% nucleotide sequence identity to the partial sequence of transposon Tn*As2*, which was identified only in *Aeromonas salmonicida* (7). This truncated form, ΔTn*As2*, was also located upstream of *mcr-3* in an *E. coli* isolate from pig feces from Malaysia, a *K. pneumoniae* isolate from a human pus sample in Thailand, and an *S. enterica* serovar Typhimurium isolate from human stool in the United States (Fig. 1B). These results suggested that the *mcr-3* gene in *Enterobacteriaceae* might have originated from *Aeromonas* species, and the Gram-negative isolates carrying *mcr-3* or *mcr-3*-like genes might already be widely disseminated in humans, animals, and the environment.

10.1128/mBio.00543-17.1 TABLE S1  Information on *mcr-3* and *mcr-3*-like genes and their deduced MCR-3 and MCR-3-like proteins collected from GenBank. Download TABLE S1, DOCX file, 0.02 MB. Copyright © 2017 Yin et al. 2017 Yin et al. This content is distributed under the terms of the Creative Commons Attribution 4.0 International license.

It should be noted that when using a breakpoint of ≥4 µg/ml, *Aeromonas* species are almost universally susceptible to colistin, except for *A. jandaei* and *A. hydrophila*. The former species seems to be intrinsically resistant to polymyxins (8), while *A. hydrophila* showed low-level resistance and exhibited induced resistance to colistin following preinduction with low concentrations (2.5 µg/ml) of colistin (9). However, the function of several *mcr-3*-like genes in different species of *Aeromonas*, including those isolated from human infections (Table S1), remains unknown and needs to be further investigated. Meanwhile, the possibility that these *mcr-3*-like genes in *Aeromonas* species have functions other than colistin resistance cannot be excluded.

In summary, we report the discovery of a new mobile colistin resistance gene, *mcr-3*, in *E. coli* of pig origin. Because of its resemblance to various other phosphoethanolamine transferases in *Enterobacteriaceae* and aeromonads identified mainly in Southeast Asia and North America in the GenBank database, this novel mobile colistin resistance gene may already be widely disseminated. Therefore, screening for the *mcr-3* gene should be urgently included in the surveillance of colistin-resistant Gram-negative pathogens from animals, humans, and the environment.

### Accession numbers.

The complete nucleotide sequence of plasmid pWJ1 has been deposited in GenBank under accession no. KY924928.
