# Erratum for Yin et al., “Novel Plasmid-Mediated Colistin Resistance Gene *mcr-3* in *Escherichia coli*”

**DOI:** 10.1128/mBio.01166-17

**Published:** 2017-08-15

**Authors:** Wenjuan Yin, Hui Li, Yingbo Shen, Zhihai Liu, Shaolin Wang, Zhangqi Shen, Rong Zhang, Timothy R. Walsh, Jianzhong Shen, Yang Wang

**Affiliations:** a Beijing Advanced Innovation Center for Food Nutrition and Human Health, College of Veterinary Medicine, China Agricultural University, Beijing, China; b The Second Affiliated Hospital of Zhejiang University, Zhejiang University, Hangzhou, China; c Department of Medical Microbiology and Infectious Disease, Institute of Infection and Immunity, Heath Park Hospital, Cardiff, United Kingdom

## ERRATUM

Volume 8, no. 3, e00543-17, 2017, https://doi.org/10.1128/mBio.00543-17. After careful review, some mistakes were found in Fig. 1A and B. These errors do not influence the major conclusions but may cause confusion. In Fig. 1A, both IS*15DI* and IS*3* are intact insertion sequences; therefore, the symbol “Δ” in the original figure indicating gene truncation was deleted from the revised version shown here. In addition, plasmid name “PWJ1” in the key showing color gradients in the right margin of panel A was incorrect and has been modified to “pWJ1.” In Fig. 1B, we did not show the whole Tn*As2* element (8,654 bp), so the former box around it has been changed to a bracket and the symbol “//” has been added to indicate that only a partial sequence of Tn*As2* is presented. Finally, both the *tnp* and *int* genes downstream of the *mcr-3*–*dgkA* segment were ignored in our previous version and have been added to both panel A and panel B.

**FIG 1:**
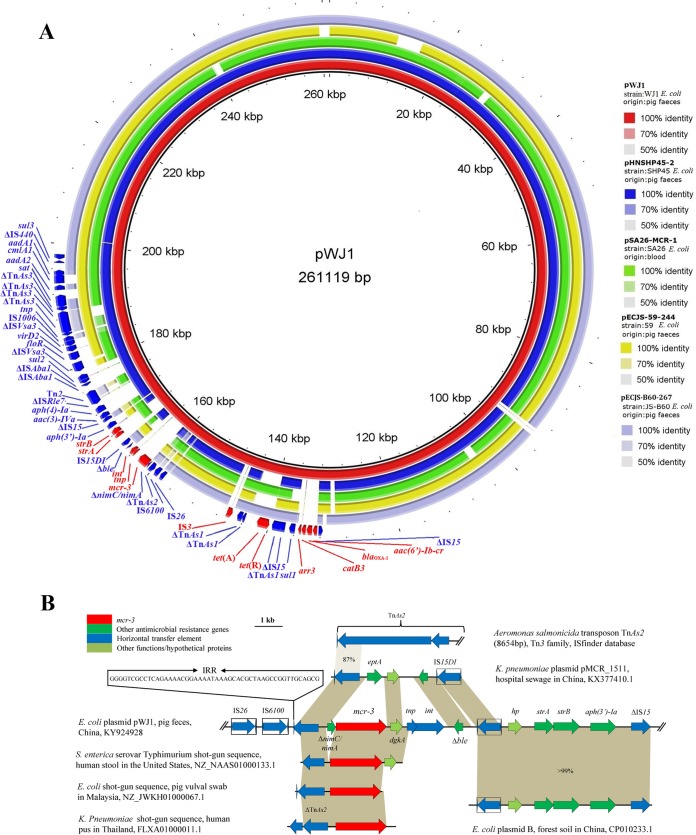
(A) BRIG analysis of the *mcr-3*-carrying plasmid pWJ1. Comparative analysis of pWJ1 with four closely related *mcr-1*-harboring plasmids from *E. coli* isolates using the BLAST Ring Image Generator. The concentric rings display similarity between the reference sequence in the inner ring and the other sequences in the outer rings. The various color levels indicate a BLAST result with a matched degree of shared regions, as shown to the right of the ring. (B) Comparison of the genetic environments of *mcr-3* genes in different plasmids and shotgun sequences extracted from the GenBank database. Arrows indicate the positions and directions of the genes; Δ indicates the truncated gene. Regions with >99% homology are indicated in gray shadow, with homology of >85% shown by a lighter gray shadow.

