# Supplementary material for: Novel Plasmid-Mediated Colistin Resistance Gene mcr-3 in Escherichia coli
Source: mBio. 2017 Jun 27;8(3):e00543-17. doi: 10.1128/mBio.00543-17 (PMC5487729; doi:10.1128/mBio.00543-17)
Supplement: TABLE S1 [file mbo003173370st1.docx]

**Table S1.** Information of *mcr-3* and *mcr-3*-like genes and their deduced MCR-3 and MCR-3-like proteins collected from GenBank database

| Strain | Nucleotide ID | Nucleotide identity with *mcr-3* | Protein ID | Protein identity with MCR-3 | Country | Sample source | Date of sample collection/data submission (yyyy/mm/dd) |
| --- | --- | --- | --- | --- | --- | --- | --- |
| *Escherichia coli* | NZ_JWKH01000067.1 | 100% | WP_039026394.1 | 100% | Malaysia | Pig vulval swab | 2013/09/16 |
| *Salmonella enterica serovar* typhimurium | NZ_NAAS01000133.1 | 100% | ORG07507.1 | 100% | United States | Human stool | 2008 |
| *Klebsiella pneumoniae* | NZ_FLWZ01000042.1 | 100% | WP_039026394.1 | 100% | Thailand | Human pus | 2015 |
| *Klebsiella pneumoniae* | NZ_FLWO01000034.1 | 99.9% | WP_065801616.1 | 99.8% | Thailand | Human urine | 2015 |
| *Klebsiella pneumoniae* | FLXA01000011.1 | 99.9% | WP_065804663.1 | 99.8% | Thailand | Human pus | 2015 |
| *Aeromonas media* | NZ_CDBZ01000089.1 | 93.9% | WP_042649074.1 | 94.8% | Unknown | Unknown | Before 2013 |
| *Aeromonas caviae* | NZ_JWJP01000016.1 | 92.7% | WP_039039919.1 | 94.1% | Malaysia | Lake water | 2014/04 |
| *Aeromonas hydrophila* | NZ_AOBN01000008.1 | 92.7% | WP_017778762.1 | 94.1% | Malaysia | Human peritoneal fluid | Before 2012 |
|  | NZ JFJO01000060.1 | 69.4% | WP_043162165.1 | 76.7% | United States | Wetland sediment | 2010/10 |
|  | NZ_LNUR01000009.1 | 69.6% | WP_060390254.1 | 76.9% | United States | Diseased Bluegill | 1997 |
|  | NZ_BAXY01000008.1 | 69.4% | WP_045527168.1 | 76.7% | Sri Lanka | Crap kidney | 2007 |
|  | JTBD01000007.1 | 69.6% | KGY49534.1 | 76.7% | United States | Human stool | 2013/10/30 |
|  | NZ_JDWB01000011.1 | 69.5% | WP_029300214.1 | 76.9% | United States | Human clinical sample | 2012/03/22 |
| *Aeromonas salmonicida* | NZ_JXTA01000057.1 | 79.7% | WP_005321527.1 | 84.5% | Switzerland | Wild brown trout | 2004 |
|  | NZ_JYFF01000058.1 | 79.7% | WP_005321527.1 | 84.5% | France | Sick fish | Before 2013 |
|  | NZ_JYFG01000055.1 | 79.7% | WP_005321527.1 | 84.5% | Switzerland | Arctic char | 2006 |
|  | NZ_LMTK01000139.1 | 79.7% | WP_005321527.1 | 84.5% | Canada | Salmo salar | 2009 |
|  | NZ_LSGV01000007.1 | 79.7% | WP_005321527.1 | 84.5% | United States | Salmo salar | 1999 |
|  | NZ_LSGX01000104.1 | 79.8% | WP_005321527.1 | 84.5% | Chile | Unknown | 2014 |
|  | NZ_MIIM01000135.1 | 79.8% | WP_005321527.1 | 84.5% | Canada | *Salvelinus fontinalis* | 2008 |
|  | NZ_MIIN01000069.1 | 79.7% | WP_005321527.1 | 84.5% | Norway | Trout | 1968 |
|  | NZ_MIIQ01000065.1 | 79.7% | WP_005321527.1 | 84.5% | Canada | *Salvelinus fontinalis* | 2009 |
|  | NZ_MIIR01000070.1 | 79.7% | WP_005321527.1 | 84.5% | Canada | *Salvelinus fontinalis* | 2010 |
|  | NZ_MIIT01000060.1 | 79.7% | WP_005321527.1 | 84.5% | United Kingdom | Salmo salar | 2000 |
| *Aeromonas sobria* | NZ_KV861273.1 | 73.5% | WP_042021679.1 | 78.4% | China | Bullfrog liver | Before 2013 |
| *Aeromonas jandaei* | NZ_JFDL01000001.1 | 72.8% | WP_041207471.1 | 78.2% | United States | River water | 2012/05/31 |
| *Aeromonas lacus* | NZ_JRGM01000144.1 | 72.6% | WP_033115156.1 | 77.8% | Finland | Recreational lake | 2013 |
| *Aeromonas piscicola* | NZ_CDBL01000034.1 | 72.6% | WP_042867079.1 | 77.6% | Unknown | Unknown | Before 2013 |
| *Aeromonas popoffii* | NZ_CDBI01000100.1 | 73.7% | WP_042039220.1 | 77.3% | Unknown | Unknown | Before 2013 |
| *Aeromonas aquatica* | NZ_CP018201 | 69.7% | WP_073350735.1 | 75.6% | China | Water | 2012/09 |
